# Tuberculous Disease of the Hip

**Published:** 1909-04-10

**Authors:** 


### Surgery.

### TUBERCULOUS DISEASE OF THE HIP.

### III.—OPERATIVE TREATMENT.

The earlier stages of this disease and their treatment have been described in recent articles, and it was there pointed out that a large percentage of cases, if diagnosed early and efficiently treated from the beginning, recover with rest and mechanical appliances, and an excellent functional result is often obtained, without any limitation of movement at the hip-joint. Unfortunately, however, this is not invariably the case, and, either because the patient has little inherent resistance to the tubercle bacillus or because treatment has been half-hearted, the disease progresses from bad to worse.

The question will then arise whether it is advisable to excise the joint or not. Some surgeons advocate early excision, without waiting for abscess or sinus formation, the argument being that in this way free access can be obtained to the joint cavity, and all the diseased material can then be removed. But it is not an operation which should be lightly advocated or undertaken. At the best the functional result is far from satisfactory, and the patient afterwards walks with a most ungainly gait, the great trochanter, which is now the highest part of the femur, slipping up and down on the dorsum ilii, and being arrested only by the attachments of the gluteal muscles to that bone. The consequence is that the patient's progression resembles that of a case of congenital dislocation of the hip. We are, therefore, strongly of opinion that all conservative methods should be given a thorough trial before recourse is had to excision of the femur.

Further than this, as Cheyne and Burghard point out, it is rarely if ever possible to remove the whole focus of disease by an open operation, and therefore it cannot be claimed that either local recurrence or general infection are guarded against by this means. Nevertheless they do think that there are certain cases in which the procedure is justifiable: these are (i) those in which the disease is obviously making active progress in spite of thorough conservative treatment, and (ii) those in which the primary disease is not in the femur but in the acetabulum, because here it is impossible to get free access to the acetabulum without previously removing the head of the femur.

In the cases which do not react to conservative treatment abscess formation is certain to occur sooner or later; and then it is not a question of whether to operate or not (since the individual cannot of his own accord get rid of caseous material), but rather of what operation should be performed, that is, whether the abscess should merely be opened and drained or whether advantage should be taken of the fact that an incision has been made to remove the head of the bone and get rid of all the diseased tissue.

Here again we are in favour of doing only so much as the circumstances of the case demand, the rationale of the procedure being, as in all cases of chronic abscess, to remove the caseous material and allow the bactericidal properties of the patient's own serum to have a chance of arresting the disease. For even although an ankylosed joint may result, such a condition is vastly preferable to the deformity resulting from excision of the head of the bone. It is, of course, good practice to remove any obviously diseased tissue and to scrape the joint cavity if this is feasible. But a question on which diverse opinions are held, and on which no such definite law can be laid down is whether any antiseptic solution should be injected into the cavity. The one most commonly advocated is iodoform emulsion. The practice has been attacked on the ground that it can be shown in the laboratory that iodoform emulsion has no active power of destroying the tubercle bacillus, and, secondly, that fatal cases of poisoning have been recorded from the absorption of this drug into the system; they are, however, admittedly rare. Solutions of corrosive sublimate used in the early days of antiseptics to be employed in this way, and disasters from this cause were not uncommon. Our own experience of iodoform emulsion goes to show that it facilitates recovery; but it is, of course, possible that this is merely a coincidence, and that the cases would have done as well without the employment of the drug.

If on opening the abscess and exploring the joint a loose sequestrum is felt, it should be removed. In advanced cases the whole head of the femur may be found lying loose in the joint cavity, and it should be taken away, since Nature has already performed an excision of the joint, and no conservative method can then prevent deformity.

In some cases the wound heals rapidly after the drainage of an abscess; but in others (and these unfortunately form the majority) a persistent sinus results. This is the outcome of one of two causes—either there is a portion of dead bone left at the bottom of the abscess cavity which acts as a chronic irritant, or else the wound has become secondarily infected at the time of operation or during subsequent dressing with one or other of the pyogenic micro-organisms. This points to imperfect technique. It is a fact which has only been sufficiently recognised in recent years that every surgical operation should be conducted with complete antiseptic precautions. The presence of an abscess, especially a chronic one, does not justify laxness in this respect. Lamentable results may occur through the superadding of a second infection.

When a sinus has formed it must be carefully probed to see if any dead bone lies at the bottom, and if any can be felt the sinus must be enlarged and the sequestrum removed. If none is found, the pus should be examined microscopically, and if a second infection is present the micro-organism present should be cultivated and the patient's resistance to it be raised by vaccine therapy. By this means the pyogenic organism can generally be eliminated, and then the sinus can be enlarged and scraped, and if care is taken with the dressings and after-treatment it ought eventually to heal. It must, however, be recognised that the treatment of abscesses and sinuses is a side-issue. The disease can only be cured by adequate rest, as outlined in the preceding articles.

